# Olfactory Dysfunction: A Complication of Diabetes or a Factor That Complicates Glucose Metabolism? A Narrative Review

**DOI:** 10.3390/jcm10235637

**Published:** 2021-11-29

**Authors:** Evanthia Gouveri, Nikolaos Papanas

**Affiliations:** 1 Diabetes Centre Dr. Tews, 63571 Gelnhausen-Hessen, Germany; vangouv@yahoo.gr; 2 Diabetes Centre, Second Department of Internal Medicine, Democritus University of Thrace, 68132 Alexandroupolis, Greece

**Keywords:** diabetes mellitus, olfactory dysfunction, diabetic complications, diabetic neuropathy, glucose metabolism

## Abstract

The present narrative review presents emerging data regarding the association between diabetes mellitus and olfactory dysfunction and discusses the role of olfactory dysfunction in glucose metabolism. We searched relevant published articles in PubMed and Google Scholar until October 2021. Main key words included “olfactory dysfunction”, “diabetes mellitus”, and “glucose metabolism”. Olfactory dysfunction has been associated with diabetes mellitus. Furthermore, it has been proposed to be a diabetic complication, given that it has been linked with microvascular complications, such as diabetic peripheral neuropathy. Interestingly, it has been suggested that olfactory dysfunction is a manifestation of central neuropathy in diabetes, a hypothesis based on the observation that diabetes, olfactory dysfunction, and cognitive decline often coexist. However, evidence is limited and inconsistent. More importantly, olfactory and endocrine systems are closely linked, and olfactory dysfunction plays a significant role in glucose metabolism and obesity. Indeed, food behaviour and energy balance are influenced by olfaction status.

## 1. Introduction

The sense of smell is very important for safety, quality of life, and metabolism [1,2]. However, it is often overlooked. Olfactory dysfunction is common [3] and it has been associated with diabetes, as well as with diabetic complications [4,5,6,7,8]. Microvascular and macrovascular mechanisms have both been proposed [4,5,6,7,8] as potential underlying pathways. Nonetheless, until now, the pathogenesis has remained to be unclear, because evidence is inconsistent. Importantly, diabetic peripheral neuropathy and neuropathic pain have been associated with olfactory dysfunction [7]. Other microvascular complications, such as diabetic retinopathy and nephropathy, were also found in association with olfactory dysfunction [4,5,8]. It has been suggested that smell tests could be used for the early diagnosis of diabetic complications [7,8]. Whether olfactory dysfunction is a microvascular or a macrovascular complication of diabetes or a manifestation of central diabetic neuropathy remains to be answered [9].

Interestingly, comorbidities in diabetes such as hepatic or renal failure, cardiovascular disease, cognitive decline, depression, and hypothyroidism have been associated with an increased risk for isolated olfactory dysfunction independent of glycaemic control [3,10]. 

Moreover, the role of olfaction in metabolism and food intake is very interesting. Data underline the role of olfactory dysfunction in quality and quantity of food intake and energy balance. The olfactory track seems to interact with hormones, such as insulin, ghrelin, and leptin which play significant roles in body weight and glucose metabolism [11] in subjects with diabetes mellitus.

The present narrative review provides an overview of the potential links between diabetes mellitus, diabetic complications, and olfactory dysfunction, and we discuss controversial issues regarding the role of olfactory dysfunction in eating behaviour and metabolic disorders. 

## 2. Search Strategy

For this narrative review, we searched relevant published articles in PubMed and Google Scholar until October 2021. The electronic search, based on the key words “olfactory dysfunction” and “diabetes mellitus”, yielded 166 articles. Articles related to COVID-19 infection were excluded. Articles addressing an association or lack of association of olfactory dysfunction with diabetes and diabetic complications were included. Furthermore, the search for the terms “olfactory dysfunction” and “glucose metabolism” yielded 87 articles. Given that we only sought to examine whether glucose metabolism affects olfaction status, not all related articles were included. Searches were not restricted by study design, but only articles published in language were included. A limitation of this review is that other databases were not used.

## 3. Olfactory Dysfunction and Diabetes Mellitus

A potential correlation of olfactory dysfunction with diabetes has been shown in previous cross-sectional studies, but results are inconsistent. Different methods to assess smell function were used, but in most studies ‘‘Sniffin’ Sticks’’ were used for the quantitative assessment of olfaction status (Table 1). Sniffin’ Sticks are odour-dispensing devices filled with 4 mL of liquid odorants and look like pens. When the cap is removed, the odour is released [12].

Le Floch et al. showed that smell recognition scores were lower in a population of 68 subjects with diabetes, type 1 diabetes mellitus (T1DM) and type 2 diabetes mellitus (T2DM) than those of 30 controls [5]. Similarly, in a cross-sectional, population-based epidemiological study among 1900 adults in Sweden, self-reported diabetes was not associated with olfactory dysfunction in 1387 adults, but it was considered to be a risk factor for anosmia (odds ratio (OR) 2.6, 95% confidence interval (CI) 1.3–5.5) [13]. Another group found no relationship between smell identification tests and diabetes in a study of 288 older African Americans using a brief smell identification test [14].

However, using Sniffin’ Sticks for the assessment of olfactory dysfunction, Brady et al. reported that all three olfactory scores (threshold, identification, and discrimination scores) were lower among 51 adults with T2DM as compared with the scores reached by 19 controls [7]. In a Greek adult population (119 subjects with T2DM and 35 controls), T2DM was independently associated with olfactory dysfunction, as evaluated by Sniffin’ Sticks. Odour threshold, odour discrimination, odour identification, and total scores were lower among subjects with T2DM (*p* < 0.001, *p* = 0.008, *p* < 0.001, *p* < 0.001, respectively) [4]. 

Furthermore, data from the U.S. National Health and Nutrition Examination Survey (NHANES) 2013–2014 with self-reported olfactory dysfunction [15] revealed a significant trend to severe olfactory dysfunctions (hyposmia and anosmia) among subjects with diabetes who received treatment (oral agents or insulin) vs. those managed with diet only (OR 1.30, 95% CI 0.59–2.90 and OR 2.87, 95% CI 1.26–6.52, respectively, *p* trend 0.01). Subjects with diabetes on insulin as compared with those without diabetes exhibited a non-significant increased prevalence of severe hyposmia/anosmia (OR 1.68, 95% CI 0.96–3.00) [15]. Interestingly, participants with diabetes treated with insulin showed a higher prevalence of phantom odours as compared with participants without diabetes (OR 2.98, 95% CI 1.41–6.32) [15]. However, no association between diabetes and prevalence of other self-reported measures of olfactory dysfunction was shown. In addition, diabetes duration was not associated with olfactory dysfunction [15]. Moreover, among older adults who participated in the same study, insulin resistance was associated with olfactory dysfunction assessed by an eight-item, self-administered scratch-and-sniff smell test. Participants with severe insulin resistance exhibited a two-fold risk of olfactory dysfunction (OR 2.25; 95% CI 1.25–4.05) as compared with subjects in the lowest Homeostasis Model Assessment for Insulin Resistance (HOMA-IR) quintile after adjustment for potential confounders. Therefore, the authors suggested that insulin resistance may play a role in olfactory dysfunction [16].

Another study included 30 subjects with diabetes and 30 controls and used the “absorbent perfumer’s paper strips” method to test the olfactory threshold [17]. There was a significant olfactory impairment among subjects with diabetes as compared with controls, but no significant association was observed between olfactory dysfunction and microvascular diabetic complications [17]. Moreover, in a population of white Australians, diabetes mellitus was among other factors (age, being male, smoking, and depression) associated with lower odour identification scores [18].

Roh et al. [19] analysed data of adults aged ≥40 years participating in the Korean National Health and Nutrition Examination Survey. Among other cardiovascular risk factors, diabetes was associated with a significantly high prevalence of olfactory dysfunction, mainly among women [19]. However, multivariate adjustment for confounding factors failed to show an association [19]. 

The only follow-up study in older adults in a 12 year follow-up has shown that decline in odour identification (assessed with Sniffin’ Sticks) was associated with age and diabetes independently of dementia and other comorbidities or risk factors [20]. 

Although data are inconsistent, the majority of studies support a relationship between T2DM and olfactory dysfunction. However, large prospective studies are needed to establish or refute a direct causal relationship. 

### 3.1. Olfactory Dysfunction and Type 1 Diabetes

The evidence on olfactory dysfunction specifically in T1DM is limited. In a cross-sectional study including 106 T1DM subjects and 30 healthy controls, no significant difference in olfactory function was observed between the 2 groups. Glycated haemoglobin (HbA_1c_) was not correlated with olfactory function assessed with Sniffin’ Sticks. However, in T1DM, the presence of neuropathy or retinopathy among T1DM was associated with lower olfactory identification scores. Olfactory function was independently associated with the presence of neuropathy. Moreover, the T1DM duration and olfactory identification score were independent predictors of neuropathy. Furthermore, the identification score was also an independent predictor of retinopathy. These results point to a relation between olfactory dysfunction and chronic microvascular complications in T1DM [22].

Another study included 39 T1DM subjects without complications and 31 healthy controls. No increase in olfactory dysfunction assessed with Sniffin’ Sticks was seen in the former. Moreover, olfactory scores were not associated with HbA_1c_ or with T1DM duration in the first group [23].

A study including 120 subjects with T1DM and 22 controls demonstrated that hyposmia was more prevalent among patients with T1DM as compared with the non-diabetic controls (70.0% vs. 45.5% respectively, *p* < 0.03). Of note, intense physical activity was associated with better olfactory function (evaluated with Sniffin’ Sticks) [24]. Similarly, Yilmaz et al. showed lower olfactory scores even among children with T1DM as compared with non-diabetic controls, although these scores ranged within the normal limits for their age [27].

### 3.2. Olfactory Dysfunction and Diabetic Complications

Diabetes mellitus has been associated with impaired sense of smell, although the perplexing question is whether diabetes per se or, in particular, diabetes with manifested complications is associated with olfactory dysfunction. It has been proposed that olfaction does not differ between uncomplicated diabetes and healthy individuals [21]. 

Several cross-sectional studies have reported an association between olfactory dysfunction and diabetic complications. In diabetes, subjects with micro- or macrovascular complications appear to have worse olfactory scores as compared with subjects free from diabetic complications [4,5,7,8].

However, there are negative studies as well [17,26,28]. Kaya et al. [28] revealed no association between odour test classification and microvascular complications (nephropathy, microalbuminuria, and retinopathy) in a study of 85 T2DM subjects. Similarly, among 3204 participants in NHANES 2013–2014 (428 subjects with diabetes), there was a significantly higher prevalence of olfactory dysfunction in subjects with diabetes vs. those without diabetes (22% vs. 15%, respectively, *p* < 0.001), but diabetic complications were not associated with olfactory dysfunction [26].

In summary, the findings are inconsistent, and this could be partly attributed to methodological differences in the assessment of olfactory status (Table 1). Olfactory dysfunction is in some studies merely self-reported, while exclusion criteria (factors that could affect olfactory status) are not clearly defined in all studies. Moreover, the small study populations, as well as the absence of prospective data render the establishment of a causal relationship impossible. Finally, the type of diabetes among participants is not always clear.

## 4. Potential Mechanisms Associating Diabetes with Olfactory Dysfunction

Data on diabetic complications and olfactory dysfunction is controversial. Some studies have found an association between the two entities implying that olfactory dysfunction could be a macro- or microvascular complication or a dysfunction of central nervous system in diabetes [4,9]. However, the potential mechanism that links diabetes mellitus with olfactory dysfunction remains unclear. 

### 4.1. Olfactory Dysfunction and Macrovascular Diabetic Complications 

Whether olfactory dysfunction is associated with macrovascular complications and cardiovascular disease (CVD) is not clear. Very limited studies have addressed this question and data remain controversial.

A U.S. study found that olfactory dysfunction may be a predictor for increased risk of CVD [29]. However, another U.S. study failed to show a significant association between CVD and olfactory dysfunction [30]. 

Weinstock et al. reported an association between macrovascular disease (coronary artery disease (CAD) and peripheral arterial disease (PAD)) in diabetes and olfactory dysfunction, suggesting that ischaemia could play a role in the pathogenesis of olfactory impairment [6]. In another study, CVD appeared to be associated with olfactory dysfunction in the entire population, by analysing data among subjects with T2DM (with the exception of odour discrimination), no association was observed between macrovascular disease (neither CAD nor PAD) and olfactory scores. Multivariate analysis showed that CVD was not an independent predictor of olfactory dysfunction [4]. 

In summary, existing data are very limited and controversial, and therefore, until now, no underlying mechanism between olfactory dysfunction and macrovascular disease in diabetes can be implied.

### 4.2. Olfactory Dysfunction and Microvascular Diabetic Complications

Few studies have reported an association between microvascular complications in diabetes and olfactory dysfunction [4,5,7], but other studies did not support this data. Methodological differences in assessment of olfactory status among different studies could be a reason for this inconsistency.

(a) Diabetic retinopathy

It has been reported that subjects with T2DM and retinopathy had significantly worse odour identification and total olfactory scores as compared with T2DM subjects without retinopathy [4]. Conversely, no association between olfactory dysfunction and retinopathy was observed in two other studies among T1DM and T2DM participants [5,8].

(b) Diabetic nephropathy

An association between microalbuminuria and lower smell recognition scores has been reported in T1DM and T2DM subjects [5]. Similarly, in another study, reduced glomerular filtration rate and increased albuminuria have been associated with olfactory dysfunction [8]. It was proposed that olfactory dysfunction could be an early indication of microvascular complications [8]. Another study, however, failed to confirm this association [4]. So far, data is very limited und inconsistent.

(c) Peripheral diabetic neuropathy

Some studies have found an association between olfactory dysfunction and peripheral diabetic neuropathy (DPN) [4,5,7]. We previously found that DPN was linked with significantly lower odour threshold, odour identification, and total olfactory scores in T2DM [4].

Diabetes has been associated with decreased olfactory sensitivity, as well as impaired olfactory discrimination and odour identification scores as compared with controls, using Sniffin’ Sticks [7]. Interestingly, lower olfactory scores have been partially attributed to presence of neuropathic pain, but the severity of pain was not associated with olfactory impairment. Importantly, subjects with T2DM without diabetic peripheral neuropathy (DPN) and subjects with T2DM and DPN but without neuropathic pain exhibited similar olfactory function to that of controls. Therefore, it was hypothesised that neuropathic pain, leading to limited concentration, could partially explain the worse olfactory scores observed among subjects with diabetic neuropathy [7]. Moreover, the authors suggested that olfactory tests among people with diabetes could help early detection of DPN [7]. 

On the contrary, other studies do not support an association between olfactory dysfunction and PDN. In a study including both T1DM and T2DM subjects, clinically manifest DPN was not associated with olfactory dysfunction [6]. Similarly, in a study including 60 T2DM subjects and 30 healthy controls, significantly higher Sniffin’ sticks and butanol threshold scores were observed among healthy controls as compared with T2DM subjects without DPN. Although subjects with DPN had lower Sniffin’ sticks scores, butanol threshold scores, and higher sucrose thresholds as compared with subjects without diabetes (*p* < 0.001, *p* < 0.001, and *p* = 0.002), no significant differences were observed among T2DM patients with and without DPN (Sniffin’ sticks scores, butanol threshold, and sucrose thresholds) [25]. 

### 4.3. Olfactory Dysfunction and Central Diabetic Neuropathy

Dysfunction of the central nervous system, cognitive decline, and depression are common in diabetes. In addition, among elderly people, the coexistence of depression and cognitive impairment has been associated with worse odour identification scores [31]. Central manifestations have been associated with peripheral and autonomic neuropathy in subjects with diabetes [9]. However, cranial neuropathies occur less frequent but are well established manifestations of diabetic neuropathy [32]. Given that olfactory dysfunction has been associated with diabetic peripheral neuropathy, it has been hypothesised that olfactory dysfunction may develop due to olfactory nerve impairment [4]. Várkonyi et al. [9] proposed that olfactory dysfunction should be regarded as a novel manifestation of central neuropathy. Moreover, due to the high cost of assessing central nervous system involvement in diabetes, a simpler method such as the assessment of olfactory dysfunction with olfactory tests could provide an easier alternative for this investigation. 

The interaction of diabetes mellitus with the olfactory system is very interesting. Emerging evidence attempts to answer the question whether diabetes affects the olfactory nerve or other areas of the olfactory tract, such as the olfactory bulb and the olfactory cortex. However, the exact underlying mechanisms remain unclear. In streptozotocin-induced diabetic rats, neurogenesis of olfactory bulb was affected leading to olfactory dysfunction [33]. Moreover, diabetes mellitus with insulin deficiency inhibits neural stem cell proliferation in the subventricular zone, leading to a decline in olfactory bulb structural plasticity. In diabetic rats, impaired olfactory sensitivity ensues [34]. Furthermore, olfactory dysfunction in T2DM rats has been associated with interleukin 1 beta (IL-1β)-mediated inflammation [35]. Lietzau et al. [36] proposed a neuronal pathology in the piriform cortex (PC) of middle-aged T2DM rats, which negatively impacted interneurons. According to the authors, this could explain olfactory dysfunction in T2DM. Piriform cortex is the largest of the olfactory cortical areas that receives direct synaptic input from the mitral and tufted cells of the olfactory bulb via the lateral olfactory tract playing a significant role in odour identification and odour quality. Interestingly, it has been shown that the identified neuropathology in PC could be counteracted by Glucagon-like peptide-1 (GLP-1)-receptor activation, proposing a potential future pharmacological target for the treatment of olfactory dysfunction in diabetes [36]. 

Smell disorders are well known in neurodegenerative diseases and loss of olfaction has been considered to be an early clinical sign of Alzheimer disease and Parkinson’s disease [37,38]. Recent studies have confirmed that there was an interplay between diabetes mellitus, olfaction, and dysfunction of the central nervous system. In a prospective study of 15 normal, 16 prediabetic, and 15 T2DM subjects, a strong association between olfactory dysfunction and specific memory impairment was observed among subjects with prediabetes and diabetes [39]. Moreover, among 151 older Japanese T2DM subjects, those with olfactory dysfunction at baseline developed dementia more frequently after 3 years than those without smell impairment [40]. These findings suggested that olfactory dysfunction in T2DM may predict development of dementia [40]. 

An analysis of data from the Korean National Health Insurance Service—National Sample Cohort during 2003–2013 [41] showed that the prevalence of olfactory dysfunction increased every year and was higher among women. In a multivariate cox regression analysis, diabetes mellitus (hazard ratio (HR) 1.976) and depression (HR 2.758) were significant risk factors for neurodegenerative disorders among subjects with olfactory dysfunction [41].

Finally, Ni et al. [42] included 36 T2DM without DPN, 28 with DPN, and 36 healthy subjects, showing for the first time that T2DM subjects with DPN had worse cognitive function with regard to memory and processing speed as compared with those without DPN. Olfactory identification was a mediating factor between nerve conduction and executive function. On the basis of these findings, the authors suggested that olfactory status might be indicative of cognitive impairment among people with DPN [42]. 

### 4.4. Olfactory Dysfunction and Glucose Metabolism

The olfactory and endocrine systems are closely connected and interact with each other. Sense of smell and odours of foods seem to play an important role in appetite, eating behaviour, and food intake, and thus in metabolism and obesity [11,43]. Olfaction seems to be modulated by hormones such as ghrelin, insulin, leptin, orexins, neuropeptide Y, and cholecystokinin [11]. It is also well established that insulin, glucagon, adrenaline, and noradrenaline play a significant role in glucose metabolism [44]. Interestingly, insulin interacts with olfactory mucosa [45].

However, evidence supporting an association between taste and smell function and energy balance is limited und inconsistent [46]. In NHANES 2013–2014, olfactory dysfunction among 428 subjects with diabetes was associated with reduced energy intake [26]. Olfactory and gustatory dysfunctions, particularly among sick people, can lead to reduced energy intake [47]. Although olfactory dysfunction has been associated with loss of appetite, no changes in dietary preferences among people with olfactory dysfunction were reported [48]. Interestingly, subjects with multiple chemosensory disorders could decrease food consumption and be more likely to lose weight, but the incidence of weight gain was highest among people with anosmia [49]. Moreover, 24-hour fasting appears to improve olfactory function [50]. Among females with extreme eating conditions, olfaction (assessed with Sniffin’ Sticks) was impaired in obese participants and increased in anorexia nervosa (hyposmia was observed in 54.3% of obese individuals and 6.4% of individuals with anorexia nervosa). Furthermore, ghrelin levels were significantly decreased in obese subjects and were related to smell impairment, suggesting that olfactory function and ghrelin could play a significant role in emotional eating and obesity [51].

Zhang et al. [52] showed that olfactory dysfunction could result in weight gain among T2DM with cognitive impairment. Among 35 people with T2DM and obesity, 35 people with T2DM without obesity, and 35 controls, obese subjects with diabetes demonstrated lower general cognition and olfactory threshold scores, decreased left hippocampal activation, and disrupted seed-based functional connectivity with right insula as compared with people with diabetes without adiposity. Interestingly, among obese people with T2DM, administration of a GLP-1 receptor antagonist for 3 months has been associated with amelioration of cognitive and olfactory function [52].

Recently, a population-based study that included 2831 adults showed that people with olfactory dysfunction had a lower mean energy consumption and a lower mean intake of total fat, protein, sodium, and potassium as compared with those of normal controls [53]. 

Similarly, in another study among 24,990 adults who participated in the Korea National Health and Nutrition Examination Survey (from 2008 to 2012), olfactory dysfunction was associated with decreased fat consumption in the total study population. In particular, among young women, olfactory dysfunction was significantly associated with reduced fat consumption and increased carbohydrate intake; among young men, it was linked with reduced protein intake [54]. In addition, in mice, a diet rich in fat seemed to decrease olfactory discrimination as a result of olfactory sensory neuron loss [55].

The effect of odours on glucose levels has been studied in rats with and without olfactory function that underwent a glucose tolerance test. Odours seemed to affect glucose ingestion-associated changes in blood glucose. For example, on the one hand, the odour of grapefruit activated the sympathetic system and depressed the blood glucose curve. On the other hand, the odour of lavender activated the parasympathetic system and resulted in more stable blood glucose levels. Importantly, the experimental damage to the olfactory mucosa and the loss of olfactory input resulted in a decrease in the area under the blood glucose curve. The authors hypothesised that the use of floral odours could contribute to a reduction in post-prandial blood glucose levels in humans [44].

Insulin resistance may influence the relationship between obesity and smell sensitivity to food odours (particularly chocolate). The higher the body mass index (BMI), the lower the odour sensitivity; the higher the insulin resistance, the lower the smell sensitivity independently of BMI. In addition, the waist-to-hip ratio seemed to also affect food odour sensitivity, supporting that hormonal and metabolic mechanisms lead to an altered olfactory perception among obese individuals [56].

In rats, there may be a bidirectional function of the olfactory system in controlling energy balance. Acute loss of smell in obese mice improved fat mass and insulin resistance. The mechanism proposed was that the sympathetic nervous system was activated through reduced olfactory input, thus, promoting lipolysis. Moreover, conditional ablation of the insulin-like growth factor 1 (IGF1) receptor in olfactory sensory neurons ameliorated olfactory function leading to obesity and insulin resistance [57].

More recently, bariatric surgery has been shown to increase smell identification, as well as to improve taste function, presumably related to an increased sensitivity to oleic acid and to 6-n-propylthiouracil [58].

Changes in eating patterns could result in diabetes and cardiovascular disease [46,49]. It now remains to be answered whether olfactory dysfunction is the cause or the result of metabolic disorders [59] and whether the modulation of olfactory stimuli with specific odours could control circulating levels of various endocrine molecules, and thus metabolic disorders such as metabolic syndrome and diabetes [57].

## 5. Conclusions 

The evidence suggests an interesting relationship between diabetes mellitus and olfactory function. An association between diabetes and olfactory dysfunction has been observed and a number of studies pointed to a link between olfactory dysfunction and diabetic complications. However, most studies were cross-sectional studies and a causal relationship could not be established. Whether olfactory dysfunction is a further microvascular complication of diabetes, or a manifestation of central neuropathy remains to be answered. More interestingly, olfactory dysfunction seems to play a significant role in food intake and energy balance, interacting with endocrine system and glucose metabolism. More studies are now warranted in order to better understand the clinical implication of this interesting interplay between diabetes and olfaction. Finally, from a practical viewpoint, more knowledge is needed on the utility of assessing olfactory function as a surrogate risk marker of diabetic complications or cognitive decline. 

## Figures and Tables

**Table 1:** Studies addressed the association of olfactory dysfunction with diabetes and diabetic complications.

Study/(First Author, Year)	Sample Size DM/C	Type of DM	Method for Assessment of OD	Association of OD with DM	OD Associated with Diabetic Complications
Le Floch et al., 1993 [5]	60 DM/30 C	T2DM and T1DM	Smell recognition score	Yes	Nephropathy, neuropathy
Weinstock et al., 1993 [6]	111 DM	73% T2DM, 27% T1DM	Odorant confusion matrix		Macrovascular disease (CAD, PAD)
Brämerson et al., 2004 [13]	1387 adults random sample (DM+C)	DM self-reported	Scandinavian odour identification test (16 odours)	DM a risk factor for anosmia	
Naka et al., 2010 [21]	76 DM/29 C	T2DM and T1DM	5-item smell identification test		Yes
Hawkins et al., 2011 [14]	63 DM/225 C older adults	T2DM	Brief smell identification test	No	
Brady et al., 2013 [7]	51 DM/19 C	T2DM	Sniffin’ Sticks (TDI)	Yes	Neuropathy with neuropathic pain
Gascón et al., 2013 [8]	61 DM	Unspecified	Barcelona smell-taste test-24		Nephropathy
Gouveri et al., 2014 [4]	119 DM/35 C	T2DM	Sniffin’ Sticks (TDI)	Yes	Retinopathy, neuropathy
Mehdizadeh Seraj et al., 2015 [17]	30 DM/30 C	Unspecified	Absorbent perfumer’s paper strips (8 concentrations)	Yes	No
Duda-Sobczak et al., 2017 [22]	106 DM/30 C	T1DM	Sniffin’ Sticks	Yes	Retinopathy, neuropathy
Altundag et al., 2017 [23]	39 DM/31 C	T1DM without complications	Sniffin’ Sticks	No	
Falkowski et al., 2017 [24]	120 DM/22 C	T1DM	Sniffin’ Sticks	Yes	
Chan et al., 2017 [15]	3151 participants (DM +C)	Unspecified	Self-reported and 8-item pocket smell test	No	
Yazla, 2018 [25]	60 DM/30 C	T2DM	Sniffin’ Sticks, butanol- and sucrose-thresholds	Yes	No difference among DM with and without neuropathy
Rasmussen et al., 2018 [26]	428 DM/2776 C		8 odours pocket smell test	Yes	No
Yilmaz et al., 2019 [27]	30 DM/30 C	T1DM- children	Paediatric smell wheel	Lower scores in DM but within normal range	
Ekström et al., 2020 [20]	1780 older adults, 7.6% DM	T2DM (only 5 with T1DM)	Sniffin’ Sticks (16-item identification)	Yes	
Turana et al., 2020 [18]	Two cohorts: 470 Indonesians, 819 white Australians	Unspecified	10-item identification test and 12-item Brief Smell	No Yes	
Kaya et al., 2020 [28]	85 DM	T2DM	Connecticut Chemosensory clinical research centre olfactory test		No

DM, diabetes mellitus; T1DM, DM Type 1; T2DM, DM Type 2; C, controls; OD, olfactory dysfunction; CAD, coronary artery disease; PAD, peripheral arterial disease; TDI, threshold-, identification-, and discrimination- scores.

## Data Availability

Data sharing is not applicable to this article as no datasets were generated or analysed during the current study.
